# Multiple Vulvar Giant Fibroepithelial Polyps: A Rare Case Occurrence

**DOI:** 10.1155/2022/5712925

**Published:** 2022-03-29

**Authors:** Andi Kurniadi, Andi Rinaldi, Herry Yulianti, Abi Ryamafi Bazar, Rose Dita Prasetyawati, Kevin Dominique Tjandraprawira

**Affiliations:** ^1^Department of Obstetrics and Gynecology, Division of Oncology Gynecology, Padjadjaran University, Bandung, Indonesia; ^2^Department of Obstetrics and Gynecology, Division of Urogynecology and Reconstructive Surgery, Padjadjaran University, Bandung, Indonesia; ^3^Department of Anatomic Pathology, Padjadjaran University, Bandung, Indonesia

## Abstract

Fibroepithelial polyps or acrochordons are benign skin tumors of mesenchymal and ectodermal origin. They are seen in 25% of the population, and their frequency increases with age. They are often found in areas with skin folds, such as the neck, axilla, submandibular, or inguinal region. However, they can also be located in the genital tract. Hormone imbalances may facilitate the development of fibroepithelial polyp s (e.g., high levels of estrogen and progesterone during pregnancy). Larger lesions are likely to arise from the proliferation of mesenchymal cells within the hormonally sensitive subepithelial stromal layer of the lower. Generally, their size does not exceed 5 millimeters. We present a 28-year-old patient with multiple giant fibroepithelial polyps with size of 20 × 12 × 8 cm and 9 × 4 × 2 cm, located on both sides of her vulva. Herein, we presented our patient along with the review of current literature pertaining to the diagnosis and the treatment of fibroepithelial polyps (FEPs) and the factors leading to excessive growth.

## 1. Introduction

Fibroepithelial polyps or acrochordons are benign skin tumors of mesenchymal and ectodermal origin. They occur in 25% of the population, and their frequency increases with age [1, 2]. These tumors vary in their clinical appearance from small, flesh-colored or hyperpigmented, papillomatous growths resembling condylomata to large pedunculated tumors that often are hypopigmented. They are usually found in skin folds, such as the neck, axilla, submandibular, or inguinal areas. However, it can be found in the genital tract, which has an epithelial structure sensitive to hormones [2, 3]. The vulvar fibroepithelial polyps generally do not exceed 5 mm, but there is literature reporting fibroepithelial polyps as long as 42 cm [2–5].

In this case report, we present a case of multiple giant fibroepithelial polyps on the vulva.

## 2. Case Illustration

A P2A0 28-year-old woman came to the Gynecologic Outpatient Clinic in Hasan Sadikin General Hospital in August 2020 with a chief complaint of a large mass on her left vulva since four years earlier. The patient first noticed an itchy swelling on her left vulva that enlarged after she delivered her 2^nd^ child four years prior, from the size of a thumb that gradually increased until its current size. There were complaints of leucorrhoea. There was no history of vaginal bleeding, trauma, sexually transmitted disease, or any medical or surgical history. Her menstrual history was unremarkable. She used hormonal contraception (three-monthly progesterone injection, followed by combined oral contraceptive pills) for three years. She was a nonsmoker and denied alcohol or drug use.

**Figure 1:**
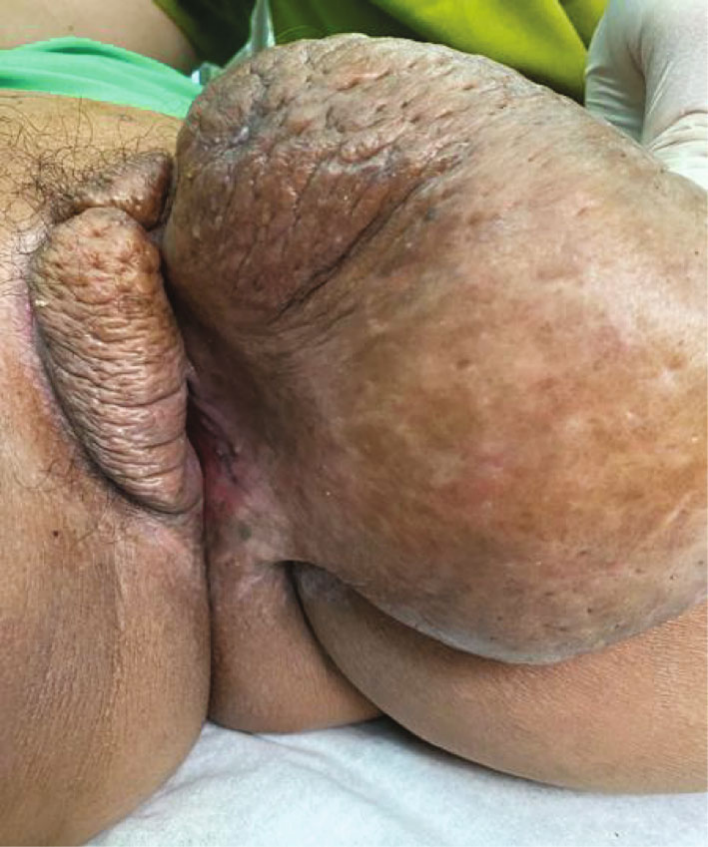
The masses in the labia.

There were no remarkable features we found from the general physical examination, except that she was obese with a BMI 36.31 kg/m^2^. On gynecological examination, we found a large, firm, nontender, nonulcerating, skin-colored pedunculated mass, measuring 15 × 10 × 8 cm protruding from the left labium majora (Figure 1). The right labium and the clitoral hood also enlarged and felt firm, sized 9 × 4 × 2 cm. Internal examination was within normal limit. The laboratory results were within normal limits. We then proceeded to do fine needle biopsy of the mass, followed by excision biopsy. The histopathology results of the biopsy was fibroepithelial polyp of the vulva. The patient was to undergo a total excision surgery of the mass.

The patient was scheduled to undergo surgery in November 2020. During her presurgical assessment, the mass had grown significantly to 20 × 12 × 8 cm.

**Figure 2:**
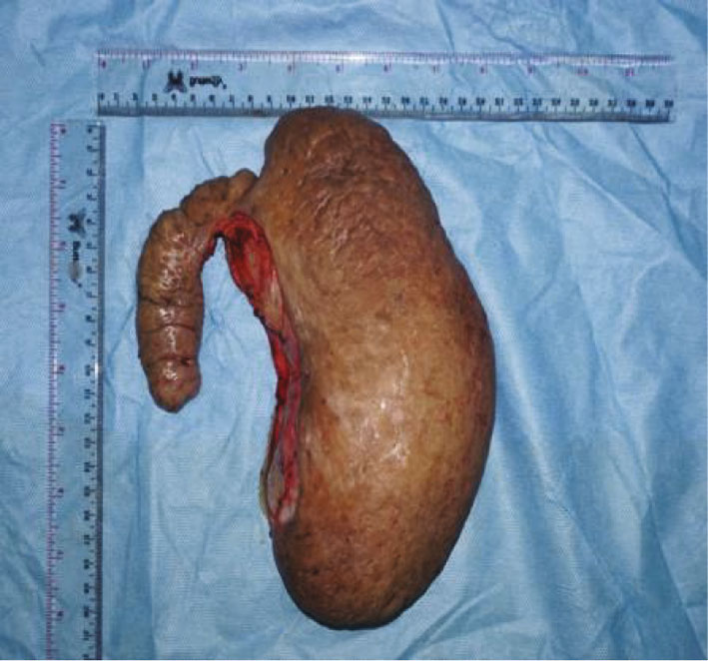
The giant fibroepithelial polyp, measuring more than 25 × 10 × 8 cm in its largest dimension.

**Figure 3:**
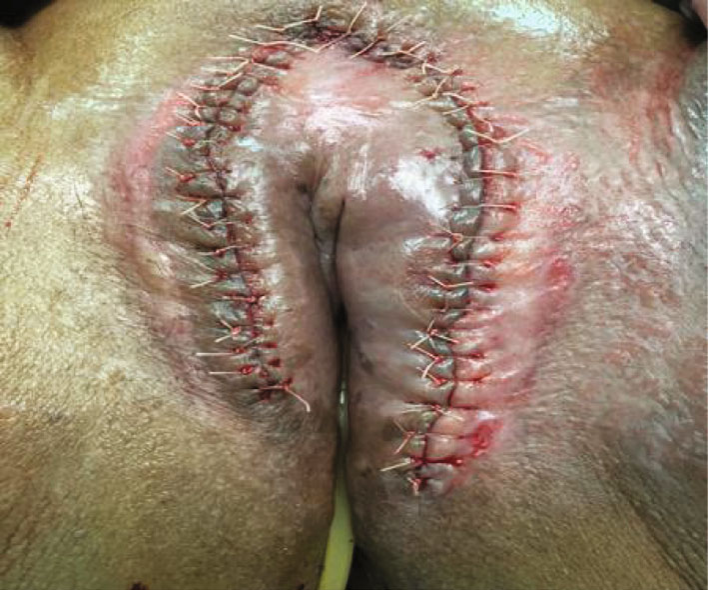
Wound closure in the vulval region.

We continued with bilateral labial excision (Figures 2 and 3). The postoperative course was uneventful. She was discharged from the hospital on her third day.

**Figure 4:**
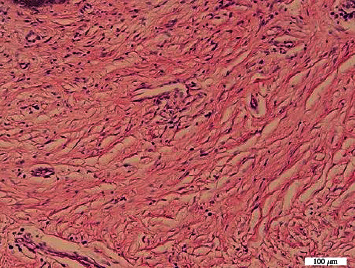
Tumor histopathology revealed the presence of keratinized and hyperplastic epithelium with underlying fibro-collagenous stroma with nonspecific inflammatory cells. No malignant cells were discovered.

The histopathologic results from the operation revealed that the epithelial surface was keratinized and hyperplastic (Figure 4). The stromal part of the fibro-collagenous tissue was fibrotic; some underwent hyalinated degeneration, as well as the presence of chronic inflammatory cells, PMN, and blood vessels. There were no malignant cells in the specimen.

The patient gave her consent for her case to be reported.

## 3. Discussion

Fibroepithelial polyp is a rare, locally infiltrative but nonmetastasizing tumor of pelvic soft tissue seen mostly in women of child-bearing age [6]. Initially, fibroepithelial polyp was described by Norris and Taylor in 1966. The first case of vulvar fibroepithelial polyp was described by Ostor et al. in 1988 [7, 8] Fibroepithelial polyps rarely arises in the vulva and cervix of reproductive-age women [9]. The most common clinical presentation of fibroepithelial polyp is a painless mass [6]. Our patient came with complaints of large mass at the left side of her vulva that gradually increased from the size of a thumb until its current size. The vulvar fibroepithelial polyps generally do not exceed 5 mm, but there is literature reporting fibroepithelial polyps as long as 42 cm [2, 4, 10]. There are a number of cases of giant fibroepithelial polyp, but vulvar mass is rare, and even rarer is a mass reaching excessive dimensions. This makes the case unique because the mass measured more than 20 cm in its widest diameter, and the lesion came on both sides of the vulva, although the lesion on the right vulva is not as big as the left one.

There are different opinions about how fibroepithelial polyps reach these sizes and what triggers the growth. The growth of fibroepithelial polyps is caused by the sensitivity of the epithelium to hormones and hormonal changes located in the genital tract and can grow to enormous sizes [2, 3, 10]. It is also remarkable that fibroepithelial polyps located in this area are seen more frequently in women and in reproductive age [10]. Our patient started to feel the complaints after delivering her 2^nd^ baby about four years ago, during which time she used hormonal contraception.

It is not known exactly what triggered the growth of the fibroepithelial polyps that are located in other locations and reached huge sizes [2]. However, a positive correlation between obesity, insulin resistance, and fibroepithelial polyp growth has been proposed [1]. In the study presenting an 18 cm long giant fibroepithelial polyp located at the axilla, it was suggested that morbid obesity of the patient might be the cause of the growth of fibroepithelial polyp [11]. Indeed, insulin resistance and obesity have also been shown in different studies as a factor for the growth of fibroepithelial polyps [12]. Another theory blames hormonal stimulation for the massive growth of such polyps [13]. Our patient started to feel the complaints after delivering her 2^nd^ baby about four years ago, during which time she used hormonal contraception. The fact that she was obese (BMI 36.31 kg/m^2^) and using hormonal contraception suggested that the two factors might have influenced the tumor growth [13].

Although the risk of malignancy and recurrence are very low, malignancy must be excluded by tissue biopsy. Botryoid embryonal rhabdomyosarcoma is the main differential diagnosis, and differentiated sarcomas from a fibroepithelial stromal polyp may be difficult [9]. A distinguishing microscopic feature would be the presence of stellate and multinucleate stromal cells, not found in sarcomas [9]. Other differential diagnoses include leiomyomas, superficial angiomyxoma, perineuroma, and neurofibromas mimic fibro epithelial polyp [14]. In the present case, definitive diagnosis was confirmed on the basis of histopathological examination. Histologically, the most characteristic feature of this polyp is the presence of stellate and multinucleate stromal cells at the epithelial-stromal interface [9]. In this case, the microscopic evaluation of the lesion revealed that the epithelial surface was keratinized and hyperplastic. The stromal part of the fibro-collagenous tissue was fibrotic; some underwent hyalinated degeneration, as well as the presence of chronic inflammatory cells, PMN, and blood vessels. There were no malignant cells in the specimen.

Ideal treatment for this lesion is complete excision and long-term follow-up to detect recurrence at the earliest. Although fibroepithelial polyps are benign skin tumors, they tend to regrow if not totally excised [9]. They can be treated with cryotherapy or cauterization when their size is in millimeters, while surgical excision is required for large fibroepithelial polyps [15]. There has been previous experience in excising giant polyps in the literature with excellent outcomes [9, 16].

## 4. Conclusion

Large fibroepithelial polyp of the vulvar region is a rare benign tumor that can be misinterpreted as malignant owing to its wide range of morphological appearances. Expert pathological interpretation may be necessary to exclude atypical tumors and malignant neoplasms or to indicate proper treatment.

## Data Availability

Research data are available upon reasonable request.
